# gauseR: Simple methods for fitting Lotka‐Volterra models describing Gause’s “Struggle for Existence”

**DOI:** 10.1002/ece3.6926

**Published:** 2020-10-26

**Authors:** Lina K. Mühlbauer, Maximilienne Schulze, W. Stanley Harpole, Adam T. Clark

**Affiliations:** ^1^ Institute of Biology Martin Luther University Halle Germany; ^2^ Department of Physiological Diversity Helmholtz Centre for Environmental Research (UFZ) Leipzig Germany; ^3^ German Centre for Integrative Biodiversity Research (iDiv) Halle‐Jena‐Leipzig Leipzig Germany; ^4^ Synthesis Centre for Biodiversity Sciences (sDiv) Leipzig Germany; ^5^ Institute of Biology Karl‐Franzens‐University of Graz Holteigasse 6, Graz, 8010 Austria

**Keywords:** competition, differential equation, growth rate, logistic growth, optimization, predator‐prey

## Abstract

Point 1: The ecological models of Alfred J. Lotka and Vito Volterra have had an enormous impact on ecology over the past century. Some of the earliest—and clearest—experimental tests of these models were famously conducted by Georgy Gause in the 1930s. Although well known, the data from these experiments are not widely available and are often difficult to analyze using standard statistical and computational tools.

Point 2: Here, we introduce the gauseR package, a collection of tools for fitting Lotka‐Volterra models to time series data of one or more species. The package includes several methods for parameter estimation and optimization, and includes 42 datasets from Gause's species interaction experiments and related work. Additionally, we include with this paper a short blog post discussing the historical importance of these data and models, and an R vignette with a walk‐through introducing the package methods. The package is available for download at github.com/adamtclark/gauseR.

Point 3: To demonstrate the package, we apply it to several classic experimental studies from Gause, as well as two other well‐known datasets on multi‐trophic dynamics on Isle Royale, and in spatially structured mite populations. In almost all cases, models fit observations closely and fitted parameter values make ecological sense.

Point 4: Taken together, we hope that the methods, data, and analyses that we present here provide a simple and user‐friendly way to interact with complex ecological data. We are optimistic that these methods will be especially useful to students and educators who are studying ecological dynamics, as well as researchers who would like a fast tool for basic analyses.

## Graphical abstract

The competition and predation experiments conducted by G. F. Gause in the first half of the 20th century remain some of the best‐known empirical tests of Alfred Lotka and Vito Volterra's models of species interactions. Here, we collate data from more than 40 of these experiments, along with basic tools for fitting the Lokta‐Volterra models to empirical data.

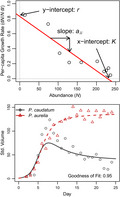

## 1 INTRODUCTION

A century ago, Alfred J. Lotka (1920, 1925) and Vito Volterra (1926) published their canonical works concerning the mathematics of species interactions. These theories were among the first to suggest that the dynamics of complex ecological systems could be described through simple underlying equations (Israel, 1988; Scudo & Ziegler, 1978). Inspired by the possibility of understanding natural systems through mathematics, Georgy Gause stated in his “Struggle for Existence” (Gause, 1934b):

[…] in order to penetrate deeper into the nature of these phenomena we must combine the experimental method with the mathematical theory, a possibility which has been created by the brilliant researches of Lotka and Volterra.

To match the strong assumptions and narrow scope of Lotka and Volterra's basic equations, Gause constructed simple experimental microcosms, in which he was able to show that interactions between pairs of species influenced their abundances and dynamics in ways that accorded with theoretical predictions (Gause, 1934a,b). These experiments laid the groundwork for a century of experiments based on mathematics as a conceptual framework. It has since become a primary goal of ecology to explain empirical observations with mathematical theory.

Despite their simplicity, the Lotka‐Volterra equations are still the primary models used to describe species interactions. At their most basic, these models describe the changes per unit time in the abundance of a species, *N_i_*, as.

(1a) $$dN_{i}/dt=N_{i}\left(r_{i}+a_{\mathrm{ii}}N_{i}+\;\Sigma_{j}\left(a_{\mathrm{ij}}N_{j}\right)\right)$$

where *r_i_* is the intrinsic growth rate, which describes the growth of a species at low density and in the absence of other species, *a_ii_* describes the effect of species *i* on its own growth, and *a_ij_* describes the effect of species *j* on the growth of species *i*. The form that we use here differs somewhat from that often portrayed in some textbooks, as we leave *r_i_* inside the parenthesis and therefore do not standardize interaction coefficients by the growth rate (thus, we signify these as *a* rather than *α*). If we divide both sides of Equation (1a) by *N_i_* to calculate the “per‐capita” growth rate of species *i*, the model can be written as a simple linear equation.

(1b) $$dN_{i}/N_{i}dt=r_{i}+a_{\mathrm{ii}}N_{i}+\;\Sigma_{j}\left(a_{\mathrm{ij}}N_{j}\right)$$

**FIGURE 1:**
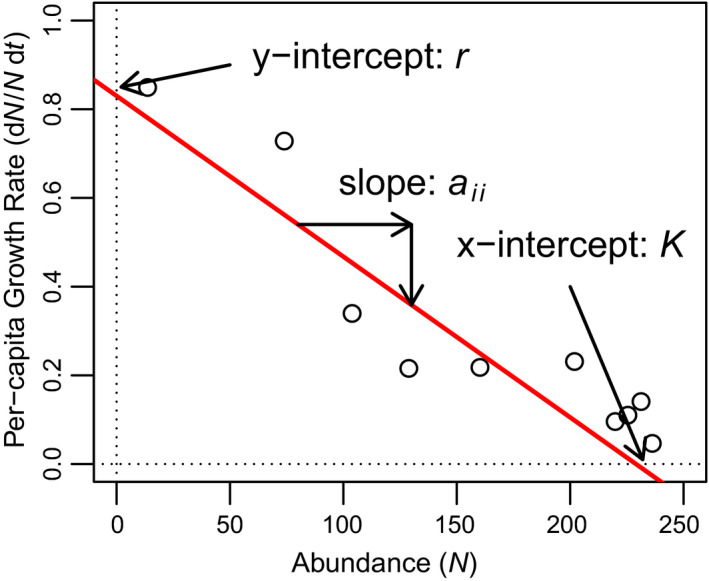
Figure comparing per‐capita growth rate (following equation 1b) versus abundance for *Paramecium aurelia* grown in monoculture. Note that when plotted in this manner, the parameter values for the Lotka‐Volterra equations can be easily “read” off the graph, which *r* as the y‐intercept, *K* as the x‐intercept, and *a_ii_* as the slope. In multi‐species systems, interaction coefficients *a_ij_* (i.e., effect of species *j* on species *i*) can be similarly computed based on the slope of d*N_i_*/*N_i_*d*t* versus *N_j_*

This form is particularly useful for analyzing empirical data, as the parameters can be estimated using ordinary least squares regression of per‐capita growth rates against species abundances, where *r_i_* is the y‐intercept, *a_ii_* is the slope with respect to species *i's* own abundance, and *a_ij_* is the slope with respect to other species (Lehman et al., 2020). Note that in a single species, this relationship can also be expressed in terms of the carrying capacity, *K_i_*, which describes the equilibrium abundance reached by a species growing in the absence of other species and falls on the x‐intercept (Figure 1). For those who are less familiar with regression or dynamical systems modeling, we have included a more in‐depth discussion of this approach in the vignette accompanying our R package. We can solve for *K_i_* in terms of *a_ii_* and *r_i_* by setting Equation (1b) equal to zero, and solving for *N_i_*, which yields.

(1c) $$K_{i}=\;‐r_{i}/a_{\mathrm{ii}}$$

Because the Lotka‐Volterra models make no specific assumptions about the mechanisms underlying species interactions, they can be parameterized in ways that approximate any combination of underlying mechanisms, at least locally around equilibrium (Chesson, 2000; Letten & Stouffer, 2019; MacArthur, 1970). For example, in a system with two species, if both *a_ij_* and *a_ji_* are less than zero, species suppress each other's growth, indicating competition. If both parameters are positive, then both species benefit from the other's presence, indicating mutualism. Alternatively, if *a_ij_* < 0 and *a_ji_* > 0, then species *i* is suppressed by species *j* whereas species *j* benefits from the presence of species *i*, indicating a predator‐prey relationship. Note, however, that in systems with more than two species, interpreting the net effects of species interactions is somewhat less straightforward—for example, if two species jointly respond to a shared predator, they may have a net negative effect on one another's abundances even if they do not interact directly (i.e., “apparent competition”, Holt (1977)).

Gause's original experimental work still provides some of the best examples of these different kinds of interspecific interactions. However, because of the limited analytical tools available to him in the early twentieth century, Gause was only able to fit models to a small subset of his experimental data. Moreover, because of the journal formatting conventions of his time, much of Gause's data was never officially published. In the years since Gause's death, his data and results have been widely used as teaching tools and are frequently cited as canonical examples of competitive and predator‐prey interactions, and have inspired theoretical work in many other fields (Takeuchi, 1996). For more details on this body of work, please see the blog post accompanying this article. Many groundbreaking studies have also gone on to reanalyze parts of his experimental data and extend some components of his theoretical models (e.g., Vandermeer, 1969). Nevertheless, to this day, only a small portion of Gause's experimental data has ever been quantitatively analyzed using the Lotka‐Volterra equations.

Here, we introduce the gauseR package, which includes a collection of functions for confronting empirical observations with the classical Lotka‐Volterra interaction models. The package combines methods for data formatting, model simulation, and parameter estimation into a simple‐to‐use, automated wrapper function. The package also acts as a digital repository for Gause's experiments on the “Struggle for Existence” and includes all available data from his 1934 book and associated publications. Below, we introduce the functions and basic workflow for the package, and explain the underlying theory. To demonstrate the package's functionality, we analyze two well‐known datasets from Gause (1934a), as well as classic examples of multi‐species interactions from McLaren and Peterson (1994) and Huffaker et al. (1963). Additionally, we discuss some limitations and possible extensions of the optimization methods used in the package. We foresee that gauseR will be especially helpful for researchers who seek simple tools for analyzing their data, as well as a teaching aid for introducing students to the Lotka‐Volterra equations and to Gause's experiments.

## 2 METHODS

### 2.1 Functions and workflow

The general purpose of the functions in the gauseR package is to fit the general Lotka‐Volterra model in Equation ((1a), (1b)) to observational time series data of one or more interacting species. The package, along with a walk‐through vignette, is available at github.com/adamtclark/gauseR. Functions are described in detail in Table 1. In general, this procedure will require estimating values for three types of parameters: starting abundances (*N_0i_*), intrinsic growth rates (*r_i_*), and interaction coefficients (*a_ij_*).

**TABLE 1:** Brief descriptions of the gauseR functions

Function	Description
get_lag	Calculates time‐lagged observations for variable x, separated by treatment.
percap_growth	Calculates per‐capita growth rate, using log ratios.
get_logistic	Calculates logistic growth for a population.
lv_interaction	Calculates d*n*/d*t* for *n* species in a Lokta‐Volterra system.
lv_interaction_log	Calculates d*n*/d*t* for *n* species in a Lokta‐Volterra system, with log‐transformed abundances. This form can be helpful for optimization routines where species abundances are close to zero.
lv_optim	Identifies optimal parameter values for a Lotka‐Volterra interaction system by simulating dynamics using differential equations.
gause_wrapper	Automatically runs routine for finding starting values and optimal parameter values for a Lotka‐Volterra interaction system.
test_goodness_of_fit	Generates an *R* ^2^‐like goodness of fit index, based on the squared difference between observed and predicted values. Index values of 1 indicate a very close fit, whereas values near or below zero indicate a poor fit.
ode_prediction	Calculates expected abundances for all *n* species in a community. This function is potentially useful in combination with other optimizer software, for example, as might be used for hypothesis testing.

For a single species system, dynamics can be directly fit to the Logistic growth model using the get_logistic function. For systems with two or more species, fitting proceeds in four steps. First, time‐lagged abundances for each species are calculated using the get_lag function, yielding pairs of abundance values separated by a fixed time interval for each species. Second, these time‐lagged abundances are used to estimate per‐capita growth rates at different time points, using the percap_growth function. This function approximates dynamics following the linearized equation.

(2) $$dN/Ndt=\log\left(N\left(t_{2}\right)/N\left(t_{1}\right)\right)/\left(t_{2}‐t_{1}\right)$$

Third, linear regressions are used to test how per‐capita growth rates vary as a function of conspecific and heterospecific species abundances, from which the parameters in Equation (1b) can be estimated. Finally, because parameter estimates from linearized growth rates often do not match observations for long or complex time series, the lv_interaction and lv_optim function are used to directly simulate dynamics and tune parameters to match model outputs to observations, which can greatly improve model performance (Carrara et al., 2015; Maynard et al., 2019; Rosenbaum & Rall, 2018). All four of these steps are automated in the gause_wrapper function, which takes observed species abundances as an input, and returns fitted parameter estimates. We suggest this option for most users.

To facilitate optimization, we include two additional functions. First, the lv_interaction_log function simulates dynamics for log‐transformed species abundances. This approach is helpful in systems where abundances approach zero, which can cause integration issues (e.g., predictions of negative abundances). This function is the default for gause_wrapper. Lastly, the ode_prediction function simulates dynamics for *n* species given a set of input parameter values and returns estimates of species abundances as a single column vector. This function is potentially useful for users who wish to apply other, more sophisticated optimization routines.

### 2.2 Data and example analyses

The gauseR package includes a wide range of digitized data from Gauses’ experimental work, as well as some other well‐known multi‐species time series. Full metadata and citations for these datasets are available in the help documentation for the package. Dataset names refer to the source and figure or table from which the data are drawn. Whenever possible, data were taken from tables in Gause's publications or appendices. Otherwise, data were digitized from figures using WebPlotDigitizer (Rohatgi, 2015).

To demonstrate how to apply our functions, we also conduct example analyses using four of these datasets. First, gause_1934_science_f02_03 shows Gause's classic competition experiment between *Paramecium aurelia* and *P. caudatum*, including both species grown in monoculture and in mixture (Gause, 1934a, b). We use these data to demonstrate parameter fitting methods for logistic growth and Lotka‐Volterra competition. Full source code for these analyses is available in the help documentation for the get_logistic and lv_interaction functions. Second, gause_1934_science_f01 includes interactions between the prey species *P. caudatum* and predator species *Didinium nasutum*, with which we demonstrate the difference between estimates derived from linearized models versus. from the optimizer fit to the full simulated dynamics. These analyses are shown in the help documentation for the lv_optim function. Third, to demonstrate parameter fitting in a system with more than two species, we use mclaren_1994_f03, which summarizes dynamics of wolves (*Canis lupus*), moose (*Alces alces*), and fir trees (*Abies balsamea*) on Isle Royale from McLaren and Peterson (1994). Finally, to demonstrate a case where classic Lotka‐Volterra interaction models are unable to match observations, we analyze dynamics of the prey species *Eotetranychus sexmaculatus* and predator species *Typhlodromus occidentalis* from Huffaker's classic mite experiments (Huffaker et al., 1963). Data are available in huffaker_1963. Source code for the analyses of the McLaren and Huffaker datasets is available in the Appendix S1.

## 3 RESULTS

**FIGURE 2:**
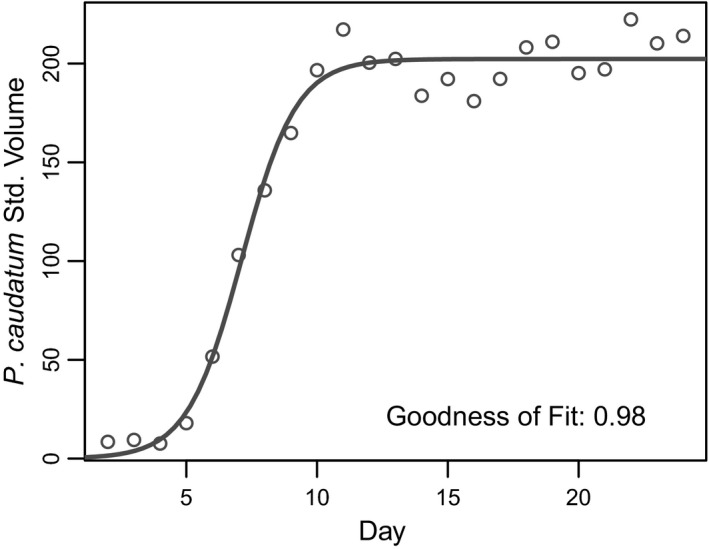
Logistic growth for size standardized volume of *Paramecium caudatum* grown in monoculture on Osterhout medium over 24 days, from Gause (1934a). Points show observations, and line shows fitted logistic growth curve, calculated with the get_log() function, that can be used to fit the logistic growth function to time series of an individual species. Goodness of fit shows results from the tests_goodness_of_fit() function. Recall that values near 1 imply a close correspondence between observations and predictions. See Table 2a for parameter values

**FIGURE 3:**
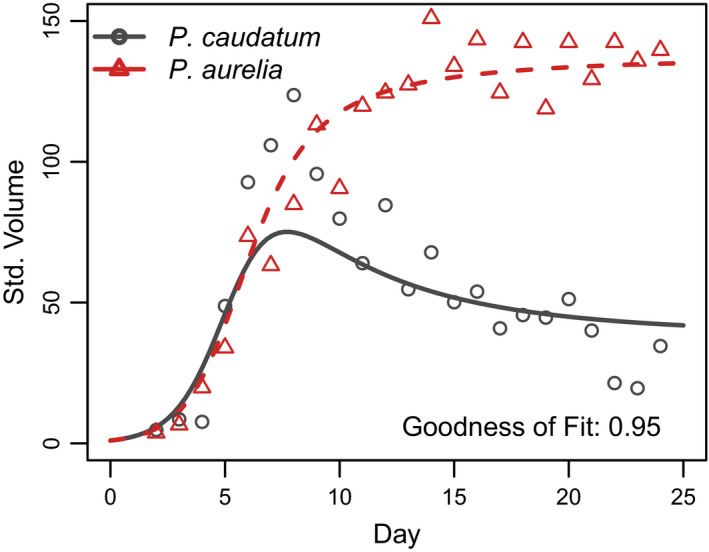
Lotka‐Volterra competition for size standardized volume of *Paramecium caudatum* and *Paramecium aurelia* grown in mixed population over 24 days, from Gause (1934a). Points show observations, and lines show growth curves fitted using the lv_interaction() function, which can be used to simulate dynamics in multi‐species mixtures following the classic Lotka‐Volterra equations. See Table 2a for parameter values

In most cases, the simple Lotka‐Volterra model in Equation ((1a), (1b)) accurately describe observed dynamics, and provide interpretable parameter values. For both logistic growth (Figure 2) and competitive interactions between the two *Paramecium* species (Figure 3), observations closely matched model predictions. Fitted parameter values suggest that both species are subject to self‐limitation in monoculture (i.e., *a_ii_* and *a_jj_* < 0) and indicate competitive interactions in mixture (i.e., *a_ij_* and *a_ji_* < 0) (Table 2a‐b).

**TABLE 2:** Fitted parameter values for each analysis described in the main text

(a)	*P. caudatum* monoculture
*N_0_*	0.22 (± 0.18)
*r*	0.96 (± 0.12)
*K*	202.31 (± 3.03)
(*a_ii_*)	−0.005 (± 0.001)

(b)	Competition experiment		
	*P. caudatum*	*P. aurelia*	
*N_0i_*	1.60 (± 1.59)	*N_0j_*	1.63 (± 1.54)
*r_i_*	1.26 (± 0.91)	*r_j_*	1.03 (± 0.55)

*A*	*P. caudatum*	*P. aurelia*
*P. caudatum*	−0.005 (± 0.015)	−0.008 (± 0.015)
*P. aurelia*	−0.002 (± 0.009)	−0.007 (± 0.008)

(c)	Predator‐prey experiment		
Linear model			
	*P. caudatum* (prey)		*D. nasutum* (predator)
*r_i_*	0.10 (± 0.30)	*r_j_*	−0.07 (± 0.54)

*A*	*P. caudatum*	*D. nasutum*
*P. caudatum*	−0.02 (± 0.01)	−0.07 (± 0.02)
*D. nasutum*	0.04 (± 0.01)	−0.03 (± 0.02)

ODE optimizer			
	*P. caudatum* (prey)		*D. nasutum* (predator)
*N_0i_*	1.66 (± 1.14)	*N_0j_*	0.12 (± 0.11)
*r_i_*	1.10 (± 0.30)	*r_j_*	−0.89 (± 0.36)

*A*	*P. caudatum*	*D. nasutum*
*P. caudatum*	−0.013 (± 0.006)	−0.08 (± 0.03)
*D. nasutum*	0.08 (± 0.02)	−0.002 (± 0.004)

(d)	McLaren and Peterson (1994)				
	*A. balsamea* (producer)		*A. alces* (herbivore)		*C. lupus* (predator)
*N_0i_*	0.33 (± 0.09)	*N_0j_*	699.90 (± 210.30)	*N_0k_*	24.31 (± 2.40)
*r_i_*	0.24 (± 0.03)	*r_j_*	2.02 (± 0.36)	*r_k_*	0.010 (± 0.008)

*A*	*A. balsamea*	*A. alces*	*C. lupus*
*A. balsamea*	−0.14 (± 0.14)	−0.0002 (± 0.00002)	0
*A. alces*	0.002 (± 0.001)	0	−0.09 (± 0.02)
*C. lupus*	0	0.00004 (± 0.000004)	−0.003 (± 0.001)

(e)	Huffaker et al. (1963)		
	*E. sexmaculatus* (prey)		*T. occidentalis* (predator)
*N_0i_*	279.14 (± 60.44)	*N_0j_*	8.45 (± 0.99)
*r_i_*	0.34 (± 0.10)	*r_j_*	−0.24 (± 0.10)

*A*	*E. sexmaculatus* (prey)	*T. occidentalis* (predator)
*E. sexmaculatus*	0	−0.06 (± 0.02)
*T. occidentalis*	0.0005 (± 0.0001)	0

	Predator self‐limitation		
	*E. sexmaculatus* (prey)		*T. occidentalis* (predator)
*N_0i_*	64.03 (± 5.78)	*N_0j_*	5.23 (± 0.60)
*r_i_*	0.187 (± 0.004)	*r_j_*	−0.38 (± 0.02)

*A*	*E. sexmaculatus*	*T. occidentalis*
*E. sexmaculatus*	0	−0.028 (± 0.003)
*T. occidentalis*	0.0012 (± 0.0001)	−0.024 (± 0.002)

Note

Values show parameter estimates ± one standard error. For interaction matrices *A*, parameters show the effect of the species listed in the *column* on the species listed in the *row* (i.e., *a_ij_* is listed in column *j* and row *i*). Note that standard errors are approximated from the numerically differentiated Hessian values calculated for the log‐transformed parameters, based on the difference between the mean estimate and the lower standard deviation in log space, and are therefore only meant as rough approximations of the degree of uncertainty. Note that values are rounded to two digits, except where needed to indicate sign.

**FIGURE 4:**
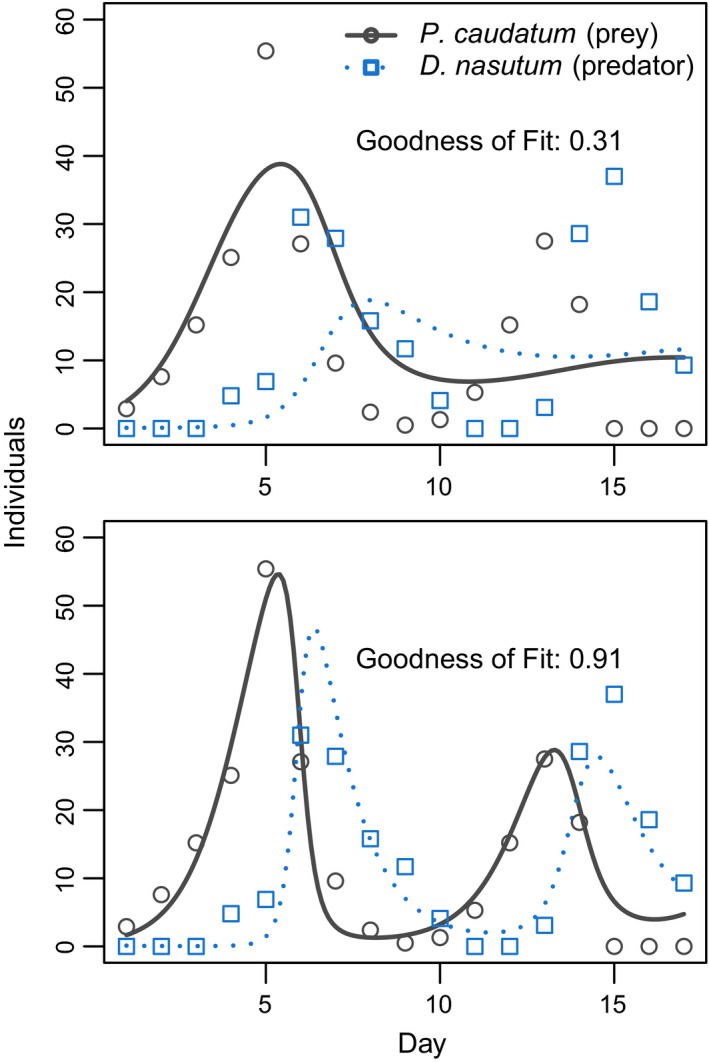
Predator‐prey interactions between *Didinium nasutum* and *Paramecium caudatum* grown in mixture over 17 days, from Gause (1934a). Additional individuals of both species were added to the mixture periodically to prevent local extinction. Points show observations, and lines show fitted growth curves. Top panel shows results for model fitted using linear regressions of species abundances versus per‐capita growth rate, whereas bottom panel shows results for parameters fitted using the simulated differential equations using the lv_optim() function. Note that goodness of fit is varies substantially between the two methods. See Table 2c for parameter values

**FIGURE 5:**
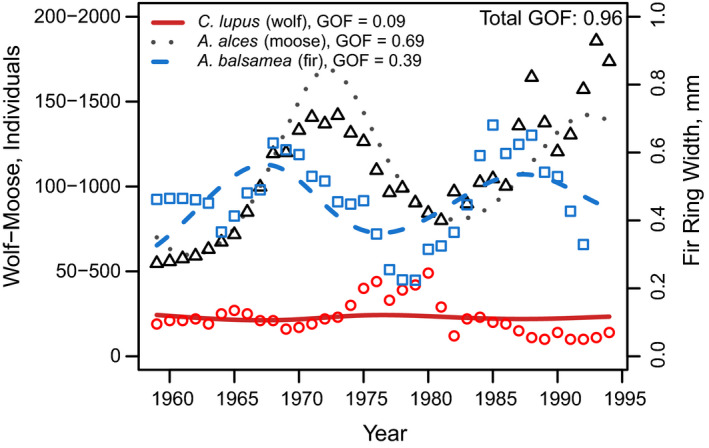
Multi‐trophic dynamics for wolves, moose, and fir trees on Isle Royale from 1960 to 1994, from McLaren and Peterson (1994). Points show observations, and lines show growth curves fitted using the lv_optim() function. The left axis shows wolf and moose abundances, separated by a ten‐fold scaling difference for easier visualization, whereas the right axis shows tree growth increments. Two methods for testing goodness of fit are shown. In the legend, values show univariate tests for each species, whereas “Total GOF” shows fit when considered across all observations simultaneously. Note that these two methods of comparison can lead to very different conclusions. See Table 2d for parameter values

For predator‐prey interactions between *D. nasutum* and *P. caudatum*, we found that parameter estimates from regressions fitted to the linearized growth rates failed to match observed dynamics (Figure 4; Table 2c). However, predictions improved substantially after using the optimizer to tune parameters. For the wolf‐moose‐fir system of McLaren and Peterson (1994), results from the optimizer also matched observed dynamics (Figure 5). Because we expected no direct interactions between wolves and fir trees, we set these interactions to zero prior to analyses. Additionally, because McLaren *et al*. hypothesized that moose populations are subject to top‐down control in this system, we set self‐limitation for moose to zero (Table 2d).

**FIGURE 6:**
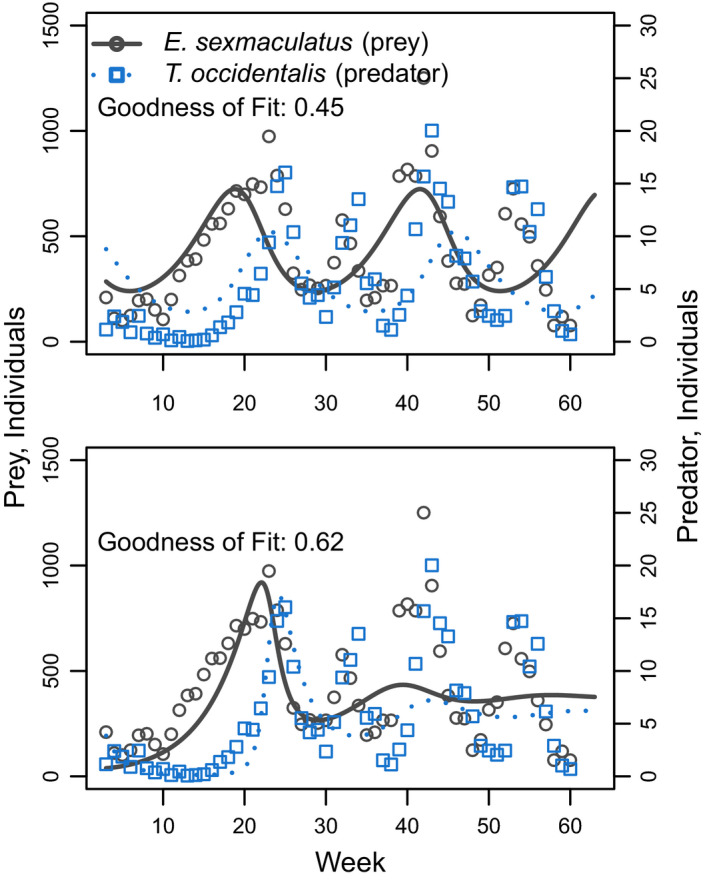
Predator‐prey interactions between *Eotetranychus sexmaculatus* and *Typhlodromus occidentalis* in a spatially structured experiment carried out on a grid of oranges over 60 weeks, from Huffaker et al. (1963). Points show observations, and lines show growth curves fitted using the lv_optim() function. Top panel shows dynamics for a system where neither species directly inhibits its own growth (i.e., *a_ii_* = *a_jj_* =0), whereas bottom panel is for a system where only the predator directly inhibits its own growth. Note that neither option is able to fully capture the realized dynamics, and that goodness of fit is relatively low in both panels. See Table 2e for parameter values

Lastly, for the Huffaker mite data, we were not able to fit the general Lotka‐Volterra model to match observed dynamics (Figure 6; Table 2e). In this system, dynamics follow sustained oscillations, but with periods and amplitudes that vary over time. However, our fitted models could only achieve either sustained oscillations of a fixed period and amplitude (top panel), or damped oscillations where the amplitude declined over time (bottom panel), but not both.

## 4 DISCUSSION

The gauseR package makes it possible to analyze time series data in single‐ or multi‐species systems using the classic Lotka‐Volterra interaction models in an easy and automated way. For almost all of the examples that we consider here, models were able to accurately capture observed dynamics, and analyses could be conducted using just the default gause_wrapper function without any modification of the code or additional inputs from the user. Furthermore, fitted parameter values matched those expected for logistic growth, competition, and predator‐prey dynamics, as predicted by Lotka and Volterra. We are therefore hopeful that this combination of fitting tools, along with the package's repository for Gause's data, will be especially useful for teaching Lotka‐Volterra's basic equations and introducing Gause's experimental work.

### 4.1 Interpreting the models

In the monoculture experiments, our results demonstrate that *P. caudatum* followed logistic growth. As expected by theory, the species was able to increase from low abundance (i.e., *r_i_* > 0), limited its own growth as its abundance increased (i.e., *a_ii_* < 0), and approached a fixed carrying capacity (*K*) over time (Gause, 1934a, b; Lotka, 1925; Volterra, 1926). For competition between *P. caudatum* and *P. aurelia*, fitted parameters indicated self‐limitation by both species, and inhibition of each species by the other (i.e., *a_ii_*, *a_jj_*, *a_ij_* and *a_ji_* < 0). Again, these values match theoretical expectations from Lotka and Volterra, and accord with Gause's original analyses of the data.

For the system with *D. nasutum* and *P. caudatum*, fitted models predicted that prey abundance increased in the absence of predators (i.e., *r_i_* > 0), and that the predators declined exponentially in the absence of prey (i.e., *r_i_* < 0,). Similarly, parameter estimates indicated that predators had a negative effect on prey, and prey had a positive effect on predators (i.e., *a_ij_* < 0 and *a_ji_* > 0, respectively). Jointly, these parameter values match biological expectations— that is, that predators consume prey, and starve when prey are absent. However, in contrast to the classic formulation of the Lotka‐Volterra predator‐prey model, we also identified self‐limitation by both species (i.e., *a_ii_* < 0 and *a_jj_* < 0). These parameter values resulted in damped oscillations in species abundances, as predicted by theory (Lehman et al., 2020). Results were similar for the wolf‐moose‐fir tree example, for which fitted parameters indicated self‐limitation by wolves and fir trees, positive effects of moose on wolves and of fir trees on moose, and negative effects of wolves on moose and of moose on fir trees. Again, predictions from the model closely matched observations, supporting the hypothesis that top‐down control of moose by wolves could explain dynamics in this system (McLaren & Peterson, 1994).

In contrast, none of the models that we fitted were able to match observations from the Huffaker *et al*. mite experiments. Importantly, this limitation is a general characteristic of the Lotka‐Volterra models and not a peculiarity of our fitting methods (Hatton et al., 2015; Lehman et al., 2020; Lotka, 1925). When self‐limitation is absent for both species, Lotka‐Volterra predator‐prey models display “neutrally stable” oscillations, meaning that oscillations maintain a fixed amplitude and period. When self‐limitation exists for either the prey species or the predator, or both, then oscillations become damped, meaning that over time they decrease in amplitude and period until they reach stable equilibrium values. Because the Huffaker data include persistent oscillations for which the amplitude and period vary over time, they cannot be matched by any parameterization of the Lotka‐Volterra equations. Potentially, these complex dynamics are a result of the spatial structure in the Huffaker experiment, which often cannot be described with simple equations (Leibold & Chase, 2018). Alternatively, they might be indicative of nonlinear or time‐varying interactions among species—for example, that result from density‐dependent interaction parameters (Murdoch, 1994). Importantly, many modern modeling tools exist that are potentially more suitable for fitting these data than are the simple Lotka‐Volterra methods that we present here (e.g., Karakoç et al., 2020).

### 4.2 Notes on optimization

For the predator‐prey examples that we analyze, our findings show that parameter estimates generated from the linearized growth rates in Equation (1b) failed to capture observed dynamics. This result accords with many other studies, which show that model performance is typically much better when parameters are tuned to match fully simulated dynamics, that is, as is accomplished with the lv_optim function (Carrara et al., 2015; Maynard et al., 2019; Rosenbaum & Rall, 2018). In general, the difference in fit between these two approaches is driven by the sensitivity of the Lotka‐Volterra equations to noisy parameter estimates—even very small changes in parameter values can lead to large changes in model predictions (Clark & Neuhauser, 2018; Maynard et al., 2019). We therefore suggest that in most cases, the gause_wrapper function, which automatically applies the optimization routine to simulated dynamics, will provide the best fits to empirical data.

Importantly, the gause_wrapper function applies several optimization “tricks” that help improve model performance. First, comparisons between observations and predictions are carried out on standardized abundances, such that the mean for each species is equal to one. This prevents abundant species from dominating the fitting process. For example, for the McLaren & Peterson data, optimization without standardization would closely fit dynamics of moose, the most common species, at the cost of model performance for wolves and fir trees. Second, by default, dynamics are simulated for log‐transformed abundances and are then back‐transformed for comparisons to observations. This approach helps prevent computational errors in the integration from erroneously driving abundances negative. Third, we also log‐transform parameter values during the optimization process, after determining their sign a priori from the linearized functions. This makes it possible to limit the number of positive interaction coefficients, which otherwise can cause the system to generate unrealistically high abundance values (e.g., via run‐away mutualism) and crash the optimizer (Lehman et al., 2020). A trade‐off that comes with this added stability is that this method does not allow for significance testing, because it constrains the sign of parameters. Thus, the standard errors provided by the gause_wrapper should only be thought of as rough approximations of the degree of uncertainty for each parameter estimate.

Because we rely on a simple OLS method for fitting models to data, our approach addresses only uncertainty that is attributable to observation error and does not consider other sources of stochasticity. There are several potential sources of observation error in the data that we analyze here. Most obviously, data digitized from figures, and especially from hand‐made figures such as those of Gause and Huffaker *et al*., are necessarily imperfect. More generally, there is reason to believe that some of Gause's original data and figures contained some errors. In particular, in a few cases where data are available in both figures and table form, there are small differences in reported values. Similarly, many of the units reported by Gause, such as “Volume” or “Amount,” are somewhat difficult to interpret and compare across figures and experiments. Lastly, in some cases, data varies between being reported for individual replicates, to mean values summed across replicates. These circumstances present a challenge in the analysis, as they make it difficult to compare values across the entire time series, and exclude information about potentially meaningful replicate‐level dynamics and between‐replicate variation. Wherever possible, we have noted these inconsistencies in the metadata.

Again, note that many more modern fitting tools and models exist that go beyond the approaches that we present here. In particular, most fitting methods that are in broad use today separately incorporate both observation error and process noise, by updating predictions based on observations in each time step (Rosenbaum & Rall, 2018; Wilkinson, 2011). These approaches have the advantage of not needing to assume that models perfectly describe deterministic interactions among species (i.e., they allow true dynamics to vary from model predictions). Additionally, many of the modeling studies since Gause apply modified versions of the Lotka‐Volterra equations, for example, by incorporating density‐dependent interaction parameters, or “higher order interactions” that vary interaction strengths as a function of community size or composition (Case & Bender, 1981; Mayfield & Stouffer, 2017; Tuck et al., 2018). Users who wish to apply more sophisticated optimization and modeling methods should see the documentation and examples for the ode_prediction function.

### 4.3 Future directions

Given the strong correspondence between the models of Lotka (1920, 1925) and Volterra (1926) and the experimental data of Gause (1934a, b), it is perhaps not surprising that these works have had such an enormous impact on ecology over the past century. Together, these concepts have helped ecologists better describe and model their systems, and have greatly advanced conceptual understanding of how different arrangements of species and interaction types are likely to play out. Nevertheless, as the type of systems that ecologists study grow ever more complex, it seems likely that we may need to move beyond these basic models to new frameworks that allow more nuanced and dynamic interaction types (Letten & Stouffer, 2019). We therefore hope that the gauseR package is useful in better exploring both the classic insights and operational limitations of Lotka, Volterra, and Gause's framework.

## CONFLICTS OF INTEREST

The authors declare no conflicts related to the research, code, or data presented in this paper.

## AUTHOR CONTRIBUTION

**Lina K. Mühlbauer:** Data curation (equal); Formal analysis (equal); Methodology (equal); Software (equal); Writing‐original draft (equal); Writing‐review & editing (equal). **Maximilienne Schulze:** Data curation (equal); Investigation (equal); Methodology (equal); Software (equal); Writing‐original draft (equal); Writing‐review & editing (equal). **W. Stanley Harpole:** Conceptualization (equal); Funding acquisition (lead); Investigation (equal); Methodology (equal); Writing‐original draft (supporting); Writing‐review & editing (equal). **Adam T. Clark:** Conceptualization (equal); Data curation (supporting); Formal analysis (equal); Investigation (equal); Methodology (equal); Project administration (lead); Software (equal); Supervision (equal); Writing‐original draft (equal); Writing‐review & editing (equal).

## Supporting information

Appendix S1

## Data Availability

All data, and corresponding metadata, for the analyses in this manuscript are available through the gauseR package, available at github.com/adamtclark/gauseR. Code, data, and metadata are available via the CRAN R package repository, as well as through Zenodo (https://doi.org/10.5281/zenodo.4058426).
