# Supplementary material for: gauseR: Simple methods for fitting Lotka‐Volterra models describing Gause’s “Struggle for Existence”
Source: Ecol Evol. 2020 Oct 26;10(23):13275–83. doi: 10.1002/ece3.6926 (PMC7713957; doi:10.1002/ece3.6926)
Supplement: Supplementary file 1 — Appendix S1 [file ECE3-10-13275-s001.zip › ece36926-sup-0003-Supinfo2.pdf]

**Blog post:**

**gauseR: Investigating Gause's "Struggle for Existence", a century after Lotka and Volterra**

If you think about the things you have learned about ecology in school, what comes to your mind? Almost certainly, there was no getting around the Lotka-Volterra equations. These equations, which have been used to describe predator-prey dynamics, competition, and interactions in general, gave you a fundamental understanding of how different species interact with one another. But, despite their ongoing relevance, it was nearly one century ago that Alfred J. Lotka and Vito Volterra were able to first set these eminent equations onto paper. Although their work eventually merged to form the singular set of equations that we know today, Lotka and Volterra originated their ideas separately.

These basic equations have been presented in many different forms and with many different parameterisations over the years. However, at their most basic, these models describe the changes per unit time in the abundance of a species, $N_i$, as

$$\mathrm{d}N_i/\mathrm{d}t = N_i(r_i + \alpha_{ii} N_i + \Sigma_j(\alpha_{ij} N_j)) \qquad \text{Eq. (1a)}$$

where $r_i$ is the intrinsic growth rate, which describes the growth of a species at low density and in the absence of other species, $\alpha_{ii}$ describes the effect of species $i$ on its own growth, and $\alpha_{ij}$ describes the effect of species $j$ on the growth of species $i$. If we simply divide both sides of Eq. (1a) by $N_i$ to calculate the "per-capita" growth rate of species $i$, the model can be written as a simple linear equation

$$\mathrm{d}N_i/N_i\mathrm{d}t = r_i + \alpha_{ii} N_i + \Sigma_j(\alpha_{ij} N_j) \qquad \text{Eq. (1b)}$$

This form is particularly useful for analyzing empirical data, as the parameters can be estimated using ordinary least squares regression of per-capita growth rates against species abundances. An example is shown in the figure below:

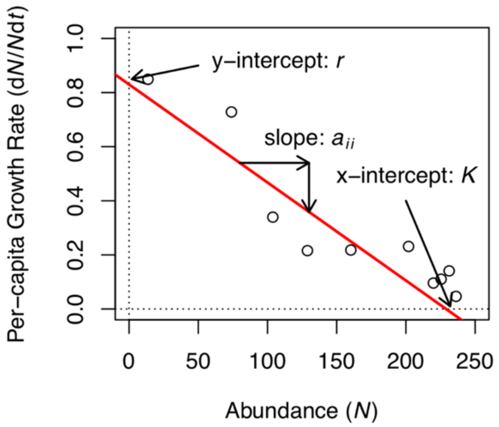

Note that $r_i$ is the y-intercept, $\alpha_{ii}$ is the slope with respect to species *i*'s own abundance, carrying capacity $K$ is the x-intercept (and can be computed as $-r_i/\alpha_{ii}$). If we were to carry out a multivariate regression, $\alpha_{ij}$ can be computed as the slope with respect to other species (Lehman *et al*. 2020). These theories were among the first to suggest that the dynamics of complex ecological systems could be described through simple underlying equations (Giorgio 1988).

<u>But how did Lotka and Volterra independently develop these equations?</u>

Initially, Alfred Lotka proposed the idea for the species interaction models in 1910. There he described autocatalytic chemical reactions and proposed to treat organisms in biological systems like molecules in chemical systems. Later on, he connected this hypothesis with biological examples in “Analytical note on certain rhythmic relations in organic systems” (1920).

Following this idea in his book “Physical elements in Biology” (1925), he started to discuss this concept in a broader evolutionary context. The exchange of energy and matter therefore happens in ecosystems through food web interactions, growth and reproduction. Starting from Darwin's natural selection principle of evolution, he stated that selection favors the maximum useful energy flow. That’s why he applied energetics and laws of thermodynamics to life sciences. Instead of creating a new discipline, which was his primary goal, he ended up laying the groundwork for the field of theoretical population ecology that we know today.

In contrast, Vito Volterra, an Italian mathematician and physicist, began with a problem laid out by Umberto D’Ancona, his son-in-law. Regarding data from fisheries in the Adriatic Sea between 1905 and 1923, D’Ancona tried to explain the increase in abundance of sharks, due to a cessation of fishing during World War I. He confronted Volterra with the problem of competition among animal species and asked him whether it might be able to explain these dynamics using a mathematical model.

Volterra’s interest in the application of mathematics to explain and explore the natural facets of complex systems was relatively novel in the non-physical sciences, especially in biology and economics, and ultimately led him to focus on cases of two-species associations. Volterra summed up these analyses in the popular three Volterra-laws, which summarized basic expectations for predator-prey dynamics, and that are known from biology lessons in school to this day. His first results studying variations and fluctuations in the number of individuals in animal species living together were published 1926.

<u>The “Lotka-Volterra model”</u>

Although the expression “Lotka-Volterra model” is commonly used to describe all basic models of species interaction resulting from the work of Lotka and Volterra, the term historically falls within a much more narrow scope, encompassing only predator-prey interactions. Whereas Lotka was mainly interested in the effect of predators on prey and parasite host interactions, Volterra focused on investigating competitive interactions. To this end, the two of them wrote letters to each other, discussing limitations and potential further advancements for their methods in many letters. In their correspondence, Lotka wrote to Volterra:

„[…] I have been particularly interested in Section 4 in which you broach the question of encounters between individuals of the two species. I believe that here is the starting point for some further developments. " (01.December 1926)

Gause's "Struggle for Existence"

The models of Lotka and Volterra  provided a starting point for empirical tests, especially those conducted by G. F. Gause, a young Soviet microbiologist, who was inspired by the two researchers. He successfully tried to validate the Lotka-Volterra models in microcosm experiments using single-celled organisms such as yeast and paramecium. The Hudson Bay Company's well-known hare– lynx time series was, by this time, the only example of predator-prey dynamics in nature, which could confirm the oscillatory trends predicted by Volterra and Lotka. Gause reported in a letter to Volterra, dated 12 October 1933:

"I have recently written a book entitled "The Struggle for Existence", [...] I described various experimental verifications of your mathematical study of the struggle of life, and most of the experimental data well harmonize with your theory."

Famously, Gause's work provided some of the clearest examples of multi-species dynamics that follow the expectations for Lotka-Volterra competitive and predator-prey dynamics. Ultimately, Lotkas letter to Volterra proved prescient - and the general models that we know today, which can be used to describe any combinations of positive, negative, and neutral interactions among species, are therefore still colloquially referred to as the Lotka-Volterra equations.

Quantitative Ecology today

In the century since the seminal work by Lotka, Volterra, and Gause, it has become a primary goal of ecology to explain empirical observations with mathematical theory. Today, ecologists worldwide still broadly use the fundamental models of Lotka and Volterra to help guide their own experiments, as G. F. Gause did. Despite their simplicity, the models are still enormously useful for describing species interactions, and often remain some of the only general methods available for quantifying and characterising species interactions in complex, diverse systems. In many ways, the success of these models arises directly from their simplicity - because they assume no specific mechanism of interaction, they can describe an enormous range of interaction types reasonably well. As summarized by Lotka to Volterra in a 1926 letter:

"Owing to immense complexities of the physical systems presented to us in living nature it is necessary [...] to begin with modest and greatly simplified examples."

Our paper

Despite the enormous influence of Gause's experimental work on the last century of ecology, surprisingly little of his data has ever been made available in a digital format. Furthermore, despite enormous progress in ecological theory and methods, relatively few simple tools exist for fitting the Lotka-Volterra models to empirical data. To help remedy this, we are celebrating

the 100th anniversary of Lotka's seminal publication on species interactions by releasing the `gauseR` package. It contains all available data from Gause's species interaction experiments, as well as simple automated tools for fitting Lotka-Volterra models to these data. In the accompanying paper and vignette file, we demonstrate how to apply these methods, and show how they perform when fitted to  data from Gause's experiments, and several other famous ecological time series. Perhaps not surprisingly, we find that Gause's data corresponds closely to "textbook" expectations for competitive and predator-prey interactions. Jointly considering the historical background, theoretical relevance, and user friendly nature of the tools and data that we present, we hope that this package helps foster wider use and broader appreciation of the data and methods of Lotka, Volterra, and Gause. Additionally, given the increasing need for tools that are suitable for online and remote education, we are optimistic that these methods will be especially useful to students and educators who are studying ecological dynamics, as well as researchers who would like a fast tool for basic analyses.

Translations and quotes from letters included in this blog post are drawn from:

Israel, G. 1988. "On the Contribution of Volterra and Lotka to the Development of Modern Biomathematics". History and Philosophy of the Life Sciences 10:37-49.
